# Care coordination for complex cancer survivors in an integrated safety-net system: a study protocol

**DOI:** 10.1186/s12885-018-5118-7

**Published:** 2018-12-04

**Authors:** Simon J. Craddock Lee, Katelyn K. Jetelina, Emily Marks, Eric Shaw, Kevin Oeffinger, Deborah Cohen, Noel O. Santini, John V. Cox, Bijal A. Balasubramanian

**Affiliations:** 1 Department of Clinical Sciences, University of Texas Southwestern Medical Center, 5323 Harry Hines Blvd, E5.506, Dallas, TX 75390-9066 USA; 2 Harold C. Simmons Comprehensive Cancer Center, 2201 Inwood Road, Dallas, TX 75235 USA; 3 Department of Epidemiology, University of Texas Health Science Center, School of Public Health, 6011 Harry Hines Blvd, V8.112, Dallas, TX 75235 USA; 4 Department of Community Medicine, Mercer University, 1250 E. 66th St, Savannah, GA 31404 USA; 5 Department of Medicine, Division of Medical Oncology, Duke Cancer Institute and Duke University Medical Center, 20 Duke Medicine Cir, Durham, NC 27710 USA; 6 Department of Family Medicine, Oregon Health and Science Center, 3181 SW Sam Jackson Park Rd, Portland, OR 97239-3098 USA; 7 Parkland Health and Hospital System, 5201 Harry Hines Blvd, Dallas, TX 75235 USA

**Keywords:** Care coordination, Cancer survivorship care, Primary care, Oncology

## Abstract

**Background:**

The growing numbers of cancer survivors challenge delivery of high-quality survivorship care by healthcare systems. Innovative ways to improve care coordination for patients with cancer and multiple chronic conditions (“complex cancer survivors”) are needed to achieve better care outcomes, improve patient experience of care, and lower cost. Our study, Project CONNECT, will adapt and implement three evidence-based care coordination strategies, shown to be effective for primary care conditions, among complex cancer survivors. Specifically, the purpose of this study is to: 1) Implement a system-level EHR-driven intervention for 500 complex cancer survivors at Parkland; 2) Test effectiveness of the strategies on system- and patient-level outcomes measured before and after implementation; and 3) Elucidate system and patient factors that facilitate or hinder implementation and result in differences in experiences of care coordination between complex patients with and without cancer.

**Methods:**

Project CONNECT is a quasi-experimental implementation study among 500 breast and colorectal cancer survivors with at least one of the following chronic conditions: diabetes, hypertension, chronic lung disease, chronic kidney disease, or heart disease. We will implement three evidence-based care coordination strategies in a large, county integrated safety-net health system: 1) an EHR-driven registry to facilitate patient transitions between primary and oncology care; 2) co-locating a nurse practitioner trained in care coordination within a complex care team; 3) and enhancing teamwork through coaching. Segmented regression analysis will evaluate change in system-level (i.e. composite care quality score) and patient-level outcomes (i.e. self-reported care coordination). To evaluate implementation, we will merge quantitative findings with structured observations and physician and patient interviews.

**Discussion:**

This study will result in an evaluation toolkit identifying key model elements, barriers, and facilitators that can be used to guide care coordination interventions in other safety-net settings. Because Parkland is a vanguard of safety-net healthcare nationally, findings will be widely applicable as other safety-nets move toward increased integration, enhanced EHR capability, and experience with growing patient diversity. Our proposal recognizes the complexity of interventions and scaffolds evidence-based strategies together to meet the needs of complex patients, systems of care, and service integration.

**Trial registration:**

ClinicalTrials.gov, NCT02943265. Registered 24 October 2016.

## Background

Nearly 70% of the 13 million people living with cancer are “complex cancer survivors,” i.e., those also dealing with other chronic conditions [1, 2]. Further, the prevalence of complex cancer survivors is expected to continue growing [3–7]. Poor, under- and uninsured individuals have the highest burdens of multiple chronic conditions and this is also similarly reflected among cancer survivors [8–18]. Complex cancer survivors need highly coordinated care to ensure optimal outcomes for their cancers, co-existing chronic conditions, and overall quality of life. However, following initial cancer treatment, needs of complex cancer survivors are not well met, resulting in poor health outcomes [4]. Management of a new cancer diagnosis often interrupts existing chronic disease care because patients undergo intensive cancer care for an extended period during which attention to their other conditions may wane. Further, patients with cancer often continue to be followed by oncologists and other specialists [19–22] with little or no care coordination with primary care clinicians. As a result, care is fragmented [23] and the providers siloed, [24–26] resulting in suboptimal care quality [27].

Effective care coordination organizes patient care activities and provider information-sharing to facilitate shared-care and appropriate service delivery [21, 24, 28–30]. Interventions to improve care coordination have been tested in primary care settings for conditions such as diabetes, hypertension, and depression [31–34] and shown to be efficacious in improving health care delivery and patient outcomes. However, few studies to date have targeted complex cancer survivors to improve care coordination between oncology and primary care [21]. With the increasing number of cancer survivors (18 million by year 2022) [7], we urgently need to identify innovative ways to improve care coordination for patients with cancer and multiple chronic conditions to achieve the triple aim of better care outcomes, better patient experience of care, and lower cost [35, 36].

To address this need, our proposed study, Project CONNECT, will adapt and implement care coordination strategies, shown to be effective for primary care conditions, among complex cancer survivors in a large, county integrated safety-net health system. The evidence–based care coordination strategies include: 1) an EHR-driven registry to facilitate patient transitions between primary care and oncology care; 2) co-locating a nurse practitioner trained in care coordination within a complex care team; 3) and enhancing teamwork through coaching. We will evaluate the effectiveness of the intervention on system- and patient-level outcomes and elucidate system and patient factors that facilitate or hinder implementation using mixed methods.

### The aims of this study are

#### Aim 1

Implement a system-level EHR-driven intervention for 500 complex cancer survivors at Parkland, combining three evidence-based care coordination strategies.

#### Aim 2

Test effectiveness of the strategies on system-and patient-level outcomes using a rigorous, quasi-experimental design with outcomes measured before and after implementation.

#### Aim 3

Elucidate system and patient factors that facilitate or hinder implementation and result in differences in experiences of care coordination between complex patients with and without cancer.

## Methods

### Study design

Project CONNECT is a quasi-experimental implementation study and a mixed-method evaluation.

### Setting

The study will be conducted at Parkland Health and Hospital System (Parkland), the public, integrated safety-net system for Dallas County. Parkland disproportionately serves under- and uninsured racial and ethnic minority populations that bear high burdens of multiple chronic conditions. The Parkland system includes 12, community-oriented primary care clinics staffed with board-certified family practitioners, internists, advanced practice providers, and nurses whose demographic background align with neighborhood characteristics (60% of physicians are African American, Hispanic, or Asian, 54% female, > 50% bilingual) [37]. The Parkland primary care clinics have been NCQA-accredited Level 3 patient-centered medical homes (PCMH) since 2015. Centralized on the hospital campus, the Parkland cancer program is accredited by the *American College of Surgeons Commission on Cancer* and participates in the Texas Cancer Registry and actively engaged in quality improvement initiatives, for example, partnering with the Centers for Medicare and Medicaid Innovation in the Oncology Care Model since 2016.

### Patient population

The study includes 500 patients diagnosed with (a) Stage I-III breast or colorectal cancers and (b) diagnosed with one or more of the following chronic conditions: diabetes, hypertension, chronic lung disease, chronic kidney disease, or chronic heart disease.

### Intervention design

We will implement a system-level EHR-driven intervention for 500 complex cancer survivors at Parkland, combining three evidence-based care coordination strategies:

#### EHR-based registry to facilitate patient transitions between primary care and oncology

**Fig. 1:**
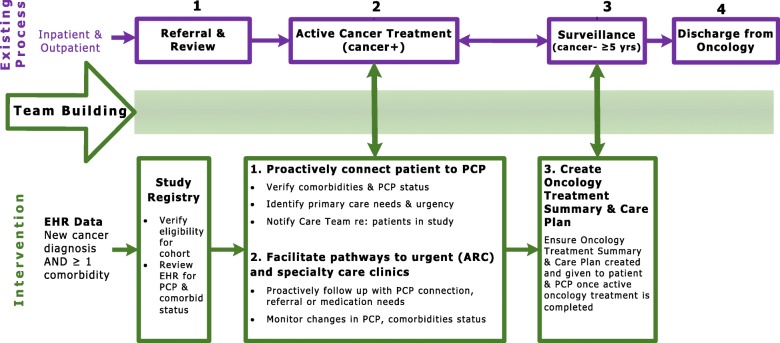
EHR-driven pathway between oncology and primary care

To facilitate transitions between oncology and primary care, we will develop an EHR-based registry and workflow [38] using the Epic Reporting Workbench to identify eligible patients and deliver team-based coordinated care (Fig. 1). The Oncology and Primary Care Medical Directors will provide clinical expertise to develop the EHR registry and associated workflow and will also champion implementation of the care coordination strategies. During Year 1, Parkland will educate clinicians and staff about the proposed system change and inform primary care physicians on the ways that the health system seeks to address this need through new patient pathways and access. This training will be included in one of each primary care clinic’s monthly training modules.

#### Co-locate a nurse coordinator trained in care coordination

Oncology will designate a full-time registered nurse (“nurse coordinator”) with competencies in care coordination [39] as a key member of the patients’ care team [40]. The nurse coordinator will be tasked primarily in clinical care coordination, continuity/transition management [41–43], and assisting patient self-management. [39, 44, 45] The nurse coordinator will review a list of patients from the registry who have initiated cancer treatment in ambulatory oncology using the EHR algorithm and confirm eligibility for this research (Fig. 1). We will confirm the algorithm’s ability to identify Stage I-III complex patients via chart review audit of 50 patients. For cancer survivors established within community primary care, the nurse coordinator will first notify the primary care physician of the survivor’s completion of initial cancer treatment and transition to the cancer survivorship phase. Patients diagnosed with cancers referred in or diagnosed through the emergency department (ED) will be assigned directly to the nurse coordinator who will establish a primary care provider and home within the Parkland system. In-person meetings at the start of cancer treatment will be established by the nurse coordinator when patients begin to attend oncology visits.

At the end of active cancer treatment, the nurse coordinator will initiate an EHR-driven treatment summary and follow-up guidelines, encourage continual interaction with primary care, and recommend transition to the care team post-treatment. S/he will systematically track patients to ascertain completion of initial cancer treatment and, each week, coordinate appointments and lab tests between primary and specialty clinics. Once appointments are made with the primary care physician and social worker, the nurse coordinator will track upcoming appointment results via the EHR-driven registry. Eligible patients will be seen again at treatment end by the nurse coordinator for a transition review. At the visit, the nurse coordinator will synthesize patients’ medical and cancer history, and educate them on follow-up for cancer recurrence, self-management for chronic diseases, and long-term effects of their cancer treatment [44]. The nurse coordinator will also coordinate appropriate specialty care referrals, including smoking cessation, and health behaviors/psychosocial counseling resources already available at Parkland. Information and education will be culturally and literacy-level appropriate, per Parkland standards, as a healthcare provider for diverse and vulnerable communities.

#### Enhance teamwork through coaching and technical assistance

An external practice facilitator will provide coaching and technical assistance to enhance team-based care [46–48]. Ongoing support from expert quality improvement trainers is critical to successfully implement and sustain system changes [41]. Prior to implementation launch, the coach will meet with the nurse coordinator in-person to start the rapport-building process and establish goals and expectations for the coordinator/coach relationship. Then, during the implementation phase, the nurse coordinator and coach will hold regular (30 min, weekly) meetings aimed at providing motivational work and direct recommendations to challenges as they arise, as well as providing specific guidance to identify and assess patients, clarify team member roles, facilitate communication, manage care transitions, and monitor or adjust work flow [49]. The external coach will draw on the principles of process improvement cycles (e.g. Plan-Do-Study-Act), appreciative inquiry, and other coaching strategies over the course of the study to help the nurse coordinator and larger team learn continuously from on-the-ground implementation [50–52].

### Evaluation design

#### Framework

We used the empirically informed *Primary Care Practice Change Model*, developed by Cohen and colleagues, to guide our mixed-methods evaluation design [53]. Built on principles of complex adaptive systems theory [53–56] and drawing from results of prior primary care system-level interventions, the model emphasizes the role of multi-level factors influencing implementation. Specifically, it identifies four key elements: internal motivators, external motivators, resources and opportunities for change [56]. These elements can act independently but, because system resources and opportunities are highly interconnected, a change in one can reverberate across the system [53]. For example, resources available to Parkland to support change (e.g. leadership and relationships among practice members) may influence the extent to which clinicians and staff adapt work flow to implement the new EHR-driven strategy to link survivors with primary care. Coupled with availability of a new care coordinator within a dedicated complex care team, this may improve care within the system. Similarly, motivated staff may identify new ways to foster implementation of the strategies. Using provider interviews and structured observations, we will examine how and why motivation of clinic members influences implementation. Using mixed-methods, we will track how these different elements inside and outside the system interact to influence change [57], and support or hamper sustainability of the intervention strategies [58–61]. We will identify system- and patient-level factors influencing implementation of the care coordination strategies to systematically pinpoint barriers amenable to ongoing quality improvement and to enable subsequent dissemination to other safety-net settings [62].

Gathering qualitative and quantitative data before, during, and after implementation will enable us to assess the factors that influence implementation at the system- and patient-levels [56, 63]. Using an intervention mixed methods approach to evaluation will enable us to observe how contextual factors shift during the course of the study [57, 64], to systematically pinpoint facilitators as well as barriers amenable to ongoing quality improvement and to generate transportable lessons for dissemination [62]. To inform real-world implementation intervention, it is important to: (a) describe how the system-level strategies are implemented; (b) elucidate contextual factors affecting implementation at system- and patient-levels; and (c) identify alternate explanations for observed effects on outcomes to generate counterfactual inference.

### Measures

Table 1 provides an overview of hypotheses, design (sample), data collection time points and outcome measures used.

**Table 1:** Hypotheses, design (sample), data collection time points, & outcome measures

Hypothesis	Design (sample)	Time points	Outcome measure (*data source*)
System-level primary hypothesis: A higher proportion of complex cancer survivors will meet quality of care guidelines for their chronic conditions and for cancer follow-up care 12 months post-implementation	Repeated cross-sections of distinct samples of patients at each time point (n~ 500)	• 12 and 24 months pre-implementation (historical controls) • 12, 24 and 36 months during implementation	Primary outcome: Composite care quality score (*EHR*) Chronic disease score - % patients receiving guideline-appropriate services (process measures) and meeting guideline-recommended targets (intermediate outcomes) Cancer follow-up care score - % patients receiving NCCN guideline-appropriate cancer surveillance
Patient-level hypothesis: Patient-reported care coordination scores will significantly improve over time among complex cancer survivors 12 months post-implementation	Repeated measures on same patients (*n* = 402)	• Baseline • 6 and 12 months post- baseline	Care coordination using patient perception of care scale [65] (*patient surveys)* - Coordination of care – overall and at visit - Specialty care access - Education/information - Preferences, social support, health literacy - Health-related quality of life

### Data collection

#### System-level data collection

##### Electronic health records (EHR) extraction

We will extract Epic EHR data to evaluate whether patients are meeting quality of care guidelines for multiple chronic conditions and follow-up National Comprehensive Cancer Network (NCCN) guidelines for cancer surveillance. A composite care quality score will be calculated for each patient as the percentage of eligible recommended services received and clinical targets met. We will also measure rates of ED utilization and hospitalizations (secondary outcomes) for ambulatory care-sensitive chronic conditions at the same time points as the primary outcomes [65, 66]. Data will be extracted 12 and 24 months before implementation and 12, 24, and 36 months during implementation.

Further, we will extract EHR data to characterize (1) visit patterns with the nurse coordinator; (2) the extent to which care coordination, disease management and referrals to specialty providers and receipt of referrals occur; (3) patients who do not receive appropriate follow-up services; (4) missed appointments or loss-to follow-up; and (5) processes related to care coordination. We will measure clinical care coordination performed by the nurse coordinator, including: number of referrals to other specialists; number of specialist appointments attended; and number of visit notes sent back to care team members’ Epic EHR inbox.

##### Structured observations

Quiet observation of daily clinical work with opportunistic discussion with staff at the primary care clinics, oncology clinics, and the complex care team will describe usual care prior to and document how workflow changes after strategies are implemented [67, 68]. Adapted from anthropological participant observation, this method lends itself to multiple levels of observation and analysis [69–72]. Research staff will conduct 40 h of structured observation prior to implementation and at 3 and 6 months post-implementation.

##### Provider interviews

Providers (*n* = 30), sampled based on their involvement in cancer survivor follow-up care and chronic disease management, will be invited to interviews assessing their perspectives on patient uptake and other challenges and opportunities. The principal investigators will develop interview guides, train and supervise the team in conducting interviews. Patients and providers completing the interview will be offered an incentive for their time and effort.

#### Patient-level data collection

##### Patient surveys

Surveys will be administered three times by phone at completion of active cancer treatment (baseline) and twice thereafter at 6 and 12 months. Patient surveys assess care coordination using the Picker Patient Perception of Care Scale [73–75], key patient-reported confounding variables (age, race/ethnicity, insurance, income, education, employment and marital status), and potential mediators and moderators (attitudes towards follow-up care, patient activation, self-efficacy, emotional and social support, health literacy, and health-related quality of life) using validated instruments [17, 76–82] (Table 2). Prior to fielding surveys, we will conduct 15 cognitive interviews (with patients who will be excluded from the larger project) to ensure measures not already validated in Spanish are adapted for language, literacy and cultural appropriateness.

**Table 2:** Patient survey constructs

Constructs	Baseline	Follow-up
Demographics	X	
Health literacy	X	
Coordination of care	X	X
Patient involvement in care	X	X
Health-related quality of life	X	X
Comorbidities	X	
Pharmacy	X	X
Self-efficacy	X	
Social support	X	
Attitudes towards follow-up		X
Depression (PHQ-9)	X	X
Patient satisfaction with navigation (PSN-L)	X	X

##### Patient interviews

Open-ended patient interviews (*n* = 70) will be conducted at baseline and throughout the 6 to 12 months post implementation of the complex care team. Both survey respondents, as well as a subset of non-respondents, will be invited to participate in a telephone interview with research staff trained in qualitative methods. Interviews will elucidate the experience of coordination for patients with multiple chronic conditions and enable us to see how cancer may create additional needs or perceptions by asking patients to talk about their experiences and communication with their provider teams. A subset of non-cancer patients with multiple chronic conditions will also be interviewed for additional comparison. Patients completing the interview will be offered a gift card for their time and effort.

### Analyses

#### System-level

We will use segmented logistic regression and linear mixed regression models to assess effect of care coordination strategies on system and patient outcomes. To evaluate system-level hypotheses, we will first use chi-square tests to compare proportion of eligible patients meeting guidelines at 12 and 24 months before implementation to proportion of patients meeting guidelines 12, 24 and 36 months after system-level strategies are in place. We will then use segmented logistic regression models [83] to identify the time point at which observed trend in proportion of patients meeting guidelines (trajectory rate) markedly changes. We will then test trend for proportion of patients meeting guidelines before implementation of system-level strategies.

##### Power

Preliminary Parkland data indicate 40% of colorectal cancer survivors (2008–10) met guidelines for cancer surveillance and chronic disease outcomes. To demonstrate a minimum increase of 10% in the primary outcome after implementation of system-level strategies as compared to before implementation, a sample of 170 patients at each time point will provide 80% power at the 0.05 significance level based on the chi-square test for comparing proportions. We have many more patients than needed; as system-level outcomes will be evaluated using EHR data, we will include all eligible patients and conduct subgroup and sensitivity analyses to better characterize effects.

#### Patient-level

For patient-level hypotheses, we will use linear mixed models to estimate effects on change in scores. Through specification of fixed effects, the mixed model allows for control of confounding variables, and can account for sources of natural heterogeneity by adding random effects that are unique to a particular individual. We will test different covariance matrices for random effects and will choose the matrix yielding the smallest Akaike information criterion or largest restricted maximum likelihood estimate. Residual analyses and plots will examine adequacy of the final model. Retrospective data might be missing. If data are missing at random (MAR), a linear mixed regression model is appropriate to address the issues of repeated measures and missing data in longitudinal studies. If the missing data are not missing at random (NMAR), we will explore the missing data pattern and a technique such as pattern mixture model will be used to adjust for NMAR bias.

##### Power

Preliminary data show mean care coordination score without intervention is 81.1 (standard deviation = 17.6) [84–86]. Assuming intra-subject correlation of 0.7 and minimum detectable standardized effect size of 0.25 (standardized effect size = effect size/standard deviation), we will need 402 patients to attain 80% power at α = 0.05 using proposed mixed-effects linear regression analyses. To accommodate 20% attrition, we will recruit 476 patients- below the 500 available in a 4-year span.

#### Mixed-method

We will combine qualitative and quantitative methods in a *convergent interactive analytic* design to examine interdependencies between conceptual model elements**.** Further, we will conduct observations at multiple time points to assess the gradual nature of implementing system change [87], staggering data collection over time, enabling each method to cross-inform our overall strategy [88]. For instance, interim analyses indicating successful implementation will inform provider interviews to elicit contextual data regarding factors contributing to increases in receipt of guideline-appropriate care [64]. Triangulating such data strengthens validity and decreases deficiencies or biases that might arise from any single method [89–91]. Our multi-disciplinary research team minimizes researcher bias by bringing together experts with differing perspectives to review and critique synthesis and interpretation of qualitative findings. By examining “factors that affect interpretation of what happened during the study (internal validity) [92] and consider what others need to know to transport the study elsewhere (external validity),” [57] our study will generate the evidence base for care coordination among complex cancer survivors and facilitate dissemination.

We will use *NVivo 9.0* (QSR International, AUS) to collate and analyze qualitative data. Use of this software makes the analytic process transparent so that investigators can track evolving analyses [93]. Research staff will transcribe field notes from structured observations and gather existing protocols and documents relating to care of complex patients to enter into the database. We will triangulate across diverse data sources, to understand how providers adapt the strategies to suit the Parkland context [56, 67]. To focus our analysis of model elements and their interdependencies, we will develop a matrix of key concepts, populating cells with observational data and inserting brief excerpts of raw text to substantiate claims or interpretations. This analytic step facilitates cross-case comparisons (e.g. pre- and post-implementation, or between patient types) and in-depth explorations of specific concepts [94]. Through monthly meetings, we will test emergent themes and interpretation against the knowledge base of our study team [95]. We will review coding agreement and resolve discrepancies through consensus [96, 97].

## Discussion

### Significance

Prior research has not addressed multiple-disease management models; the field has largely focused instead on single cancer sites or individual diseases [21, 98–101]. Our proposal recognizes the complexity of interventions and scaffolds three evidence-based strategies together to meet the needs of complex patients, systems of care, and service integration. The 2013 IOM report on high-quality cancer care calls explicitly for translation of evidence into practice and research on complex cancer survivors [102]. Our study evaluates proven intervention strategies among cancer survivors of different types contending with other multiple chronic conditions.

Although ~ 11% of cancer survivors are uninsured [4], little research has been conducted in this population who bear a disproportionate burden of multiple chronic conditions, including cancer [26, 103–106]. Similarly, care coordination strategies have mainly been implemented in healthcare systems with insured populations [107–109]. Safety-nets have important policy significance [12]: the Affordable Care Act used Medicaid expansion as a primary mechanism for extending coverage to the uninsured [10, 110]. Because community providers may not accept Medicaid, most of the new beneficiaries will likely obtain care from safety-net providers [111–113]. Finally, because Texas declined Medicaid expansion, this Dallas/Fort Worth-based study is relevant to ~ 20 non-expansion states.

### Strengths and limitations

A potential limitation of our study is the inability to evaluate using a randomized controlled trial and differential participation of survivors. However, quasi-experimental designs are uniquely suited to evaluate health services system-level interventions implemented in real-world settings [114, 115]. By incorporating additional design features such as data collection at multiple time points and a mixed-methods evaluation, we strengthen the rigor and validity of our findings by addressing history, maturation, and permit counterfactual inference [57, 92, 116]. By including two cancer types, we can evaluate potential differential effects of each care coordination strategies for different groups of survivors with multiple comorbidities. Furthermore, the use of patient-reported outcome measures makes our research patient-centered [117] and provides data on patient-level variables influencing outcomes of system-level interventions [118].

### Impact

Our intervention and mixed-methods design will result in an evaluation toolkit identifying key model elements, barriers and facilitators that can be used to guide care coordination interventions in other safety-net settings. Because Parkland is a vanguard of safety-net healthcare nationally, findings will be widely applicable as other safety-nets move toward increased integration, enhanced EHR capability, and experience with growing patient diversity [119]. Through leadership in America’s Essential Hospitals, the national association of public hospitals and health systems, Parkland will be well-positioned to provide educational programs for leaders of more than 260 member institutions and disseminate best practices in care coordination, management of multiple chronic conditions and cancer survivorship to the nation’s safety-net systems.

## Abbreviations

ED: Emergency department
EHR: Electronic health record
MAR: Missing at random
NCCN: National Comprehensive Cancer Network
NCQA: National Committee for Quality Assurance
NMAR: Not missing at random
PCMH: Patient-centered medical home
PHQ-9: Patient health questionnaire
PSN-L: Patient satisfaction with logistical aspects of navigation

## References

[1] Parry C, Kent EE, Mariotto AB, Alfano CM, Rowland JH (2011). Cancer survivors: a booming population. Cancer Epidemiol Biomark Prev.

[2] Cho H, Mariotto AB, Mann BS, Klabunde CN, Feuer EJ (2013). Assessing non-Cancer-related health status of US Cancer patients: other-cause survival and comorbidity prevalence. Am J Epidemiol.

[3] Fast Stats: An interactive tool for access to SEER cancer statistics. Surveillance Research Program, National Cancer Institute. https://seer.cancer.gov/faststats.

[4] Hewitt M, Greenfield S, Stovall E (2005). From cancer patient to cancer survivor: lost in translation.

[5] Oeffinger KC, McCabe MS (2006). Models for delivering survivorship care. J Clin Oncol.

[6] Miller S (2009). Handbook of cancer control and behavioral science: a resource for researchers, practitioners, and policy makers.

[7] Anderson G (2010). Chronic care: making the case of ongoing care.

[8] Cunningham P, Felland L, Stark L (2012). Safety-net providers in some US communities have increasingly embraced coordinated care models. Health Aff.

[9] Quinn MT, Gunter KE, Nocon RS, Lewis SE, Vable AM, Tang H (2013). Undergoing transformation to the patient centered medical home in safety net health centers: perspectives from the front lines. Ethn Dis.

[10] Ku L, Shin P, Regenstein M, Mead H. Promoting the integration and coordination of safety-net health care providers under health reform: Key issues. Issue Brief (Commonwealth Fund). 2011;22:1–13.

[11] Anderson R, Pickens S (2010). Supporting cancer services. Tex Med.

[12] Anderson RJ, Boumbulian PJ, Pickens SS (2004). The role of U.S. public hospitals in urban health. Acad Med Spec Themes: Urban Health.

[13] Agency for Healthcare Research and Quality (2012). National Healthcare Disparities Report.

[14] National Cancer Institute NIH. N.C.I. Fact Sheet: Cancer Health Disparities [Webpage]. Bethesda: National Institutes of Health; 2008. [updated 03/29/2018. Available from: https://www.cancer.gov/about-cancer/understanding/disparities].

[15] Palmer Nynikka R.A., Kent Erin E., Forsythe Laura P., Arora Neeraj K., Rowland Julia H., Aziz Noreen M., Blanch-Hartigan Danielle, Oakley-Girvan Ingrid, Hamilton Ann S., Weaver Kathryn E. (2014). Racial and Ethnic Disparities in Patient-Provider Communication, Quality-of-Care Ratings, and Patient Activation Among Long-Term Cancer Survivors. Journal of Clinical Oncology.

[16] Schootman M, Deshpande A, Pruitt S, Aft R, Jeffe D (2010). National estimates of racial disparities in health status and behavioral risk factors among long-term cancer survivors and non-cancer controls. Cancer Causes Control.

[17] Paskett ED, Alfano CM, Davidson MA, Andersen BL, Naughton MJ, Sherman A (2008). Breast cancer survivors' health-related quality of life. Cancer.

[18] Singh G, Jemal A. Socioeconomic and racial/ethnic disparities in Cancer mortality, incidence, and survival in the United States, 1950-2014: over six decades of changing patterns and widening inequalities. J Environ Public Health 2017;2017:19. 10.1155/2017/2189372. 10.1155/2017/2819372 PMC5376950 28408935

[19] Chen Chang-I, Kuan Ching-Feng, Miser James, Fang Yu-Ann, Lam Carlos, Chiu Wen-Ta, Li Yu-Chuan (2013). Comorbidity as an Independent Risk Factor in Patients With Cancer. Asia Pacific Journal of Public Health.

[20] Adams E, Boulton M, Rose PW, Lund S, Richardson A, Wilson S (2012). A qualitative study exploring the experience of the partners of cancer survivors and their views on the role of primary care. Support Care Cancer.

[21] Hudson MM, Landier W, Ganz PA (2011). Impact of survivorship-based research on defining clinical care guidelines. Cancer Epidemiol Biomark Prev.

[22] Adams E, Boulton M, Rose P, Lund S, Richardson A, Wilson S (2011). Views of cancer care reviews in primary care: a qualitative study. Br J Gen Pract.

[23] Skolarus TA, Zhang Y, Hollenbeck BK (2012). Understanding fragmentation of prostate cancer survivorship care. Cancer.

[24] O’Malley AS, Reschovsky JD (2011). Referral and consultation communication between primary care and specialist physicians: finding common ground. Arch Intern Med.

[25] Stange KC (2009). The problem of fragmentation and the need for integrative solutions. Ann Fam Med.

[26] McCabe MS, Bhatia S, Oeffinger KC, Reaman GH, Tyne C, Wollins DS (2013). American Society of Clinical Oncology statement: achieving high-quality cancer survivorship care. J Clin Oncol.

[27] Roy CL, Poon EG, Karson AS, Ladak-Merchant Z, Johnson RE, Maviglia SM (2005). Patient safety concerns arising from test results that return after hospital discharge. Ann Intern Med.

[28] McDonald K, Schultz E, Albin L, Piñeda N, Lonhart J, Sundaram V, et al. Care coordination measures atlas. Rockville: Agency for Healthcare Research and Quality; 2011. [Available from: https://innovations.ahrq.gov/qualitytools/care-coordination-measures-atlas]

[29] Enthoven AC (2009). Integrated delivery systems: the cure for fragmentation. Am J Manag Care.

[30] Care Coordination. Content last reviewed August 2018. Agency for Healthcare Research and Quality, Rockville, MD. http://www.ahrq.gov/professionals/prevention-chronic-care/improve/coordination/index.html.

[31] Rothman AA, Wagner EH (2003). Chronic illness management: what is the role of primary care?. Ann Intern Med.

[32] Vouri SM, Shaw RF, Waterbury NV, Egge JA, Alexander B (2011). Prevalence of achievement of A1c, blood pressure, and cholesterol (ABC) goal in veterans with diabetes. J Manag Care Pharm.

[33] Unützer J, Katon W, Callahan CM (2002). Collaborative care management of late-life depression in the primary care setting: a randomized controlled trial. JAMA.

[34] Coleman K, Austin BT, Brach C, Wagner EH (2009). Evidence on the chronic care model in the new millennium. Health Aff.

[35] Stiefel M, Nolan K (2013). Measuring the triple aim: a call for action. Popul Health Manag.

[36] Berwick DM, Nolan TW, Whittington J (2008). The triple aim: care, health. And Cost Health Affairs.

[37] Pickens S, Boumbulian P, Anderson RJ, Ross S, Phillips S (2002). Community-oriented primary Care in Action: a Dallas story. Am J Public Health.

[38] O’Malley Ann S., Draper Kevin, Gourevitch Rebecca, Cross Dori A., Scholle Sarah Hudson (2015). Electronic health records and support for primary care teamwork. Journal of the American Medical Informatics Association.

[39] Haas S, Ann Swan B, Haynes T (2013). Developing ambulatory care registered nurse competencies for care coordination and transition management. Nurs Econ.

[40] Shaw EK, Howard J, West DR, Crabtree BF, Nease DE, Tutt B (2012). The role of the champion in primary care change efforts: from the state networks of Colorado ambulatory practices and partners (SNOCAP). J Am Board Fam Med.

[41] Craig C, Eby D, Whittington J (2011). Care coordination model: better care at lower cost for people with multiple health and social needs.

[42] Coleman E, Parry C, Chalmers S, Min S (2006). The care transitions intervention: results of a randomized controlled trial. Arch Intern Med.

[43] Wagner EH (2012). Improving care for complex patients: the role of the R.N. care manager.

[44] Von Korff M, Gruman J, Schaefer J, Curry SJ, Wagner EH (1997). Collaborative Management of Chronic Illness. Ann Intern Med.

[45] Heijmans M, Waverijn G, Rademakers J, van der Vaart R, Rijken M (2015). Functional, communicative and critical health literacy of chronic disease patients and their importance for self-management. Patient Educ Couns.

[46] Balasubramanian BA, Chase SM, Nutting PA, Cohen DJ, Strickland PA, Crosson JC (2010). Using learning teams for reflective adaptation (ULTRA): insights from a team-based change management strategy in primary care. Ann Fam Med.

[47] Balasubramanian BA, Cohen DJ, Clark EC, Isaacson NF, Hung DY, Dickinson LM (2008). Practice-level approaches for behavioral counseling and patient health behaviors. Am J Prev Med.

[48] MacColl Center for Health Care Innovation at Group Health Research Institute. Coach Medical Home: Coaching Curricula: MacColl Center for Health Care Innovation, Commonwealth Fund, Qualis Health; 2013/ [Available from: http://www.coachmedicalhome.org/coaching-curricula].

[49] Grace SM, Rich J, Chin W, Rodriguez HP (2014). Flexible implementation and integration of new team members to support patient-centered care. Healthcare.

[50] Riggall VK, Smith CM (2015). Creating a sustainable, interprofessional-team training program: initial results. Clin Nurse Spec.

[51] Plonien C, Williams M (2015). Stepping up teamwork via TeamSTEPPS. AORN J.

[52] Vertino KA (2014). Evaluation of a TeamSTEPPS initiative on staff attitudes toward teamwork. J Nurs Adm.

[53] Cohen D, McDaniel RR, Crabtree BF, Ruhe MC, Weyer SM, Tallia A (2004). A practice change model for quality improvement in primary care practice. J Healthc Manag.

[54] McDaniel RR, Jordan ME, Fleeman BF (2003). Surprise, surprise, surprise! A complexity science view of the unexpected. Health Care Manag Rev.

[55] McDaniel R, Driebe DJ (2001). Complexity science and health care management. Adv Health Care Manag.

[56] Ruhe MC, Carter C, Litaker D, Stange KC (2009). A systematic approach to practice assessment and quality improvement intervention tailoring. Qual Manag Health Care.

[57] Stange KC, Glasgow RE. Contextual Factors: The importance of considering and reporting on context in research on the patient-centered medical home. Rockville MD: Agency for Healthcare Research & Quality; 2013 May 2013. Contract No.: AHRQ No.13–0045-EF.

[58] Crabtree BF, Miller WL, Stange KC (2001). Understanding practice from the ground up. J Fam Pract.

[59] Crabtree BF, Miller WL, Aita VA, Flocke SA, Stange KC (1998). Primary care practice organization and preventive services delivery: a qualitative analysis. J Fam Pract..

[60] Miller WL, Crabtree BF, McDaniel R, Stange KC (1998). Understanding change in primary care practice using complexity theory. J Fam Pract..

[61] Miller WL, McDaniel RR, Crabtree BF, Stange KC (2001). Practice jazz: understanding variation in family practices using complexity science. J Fam Pract..

[62] Oeffinger KC, van Leeuwen FE, Hodgson DC (2011). Methods to assess adverse health-related outcomes in cancer survivors. Cancer Epidemiol Biomark Prev.

[63] Reid RJ, Fishman PA, Yu O, Ross TR, Tufano JT, Soman MP (2009). Patient-centered medical home demonstration: a prospective, quasi-experimental, before and after evaluation. Am J Manag Care.

[64] Allen D (2013). Understanding context for quality improvement: artefacts, affordances and socio-material infrastructure. Health.

[65] Xing J, Goehring C, Mancuso D (2015). Care coordination program for Washington state Medicaid enrollees reduced inpatient hospital costs. Health Aff.

[66] Bayliss EA, Ellis JL, Shoup JA, Zeng C, McQuillan DB, Steiner JF (2015). Effect of continuity of care on hospital utilization for seniors with multiple medical conditions in an integrated health care system. Ann Fam Med.

[67] Cohen DJ, Crabtree BF, Etz RS, Balasubramanian BA, Donahue KE, Leviton LC (2008). Fidelity Versus Flexibility: Translating Evidence-Based Research into Practice. Am J Prev Med.

[68] Jaén CR, Crabtree BF, Palmer RF, Ferrer RL, Nutting PA, Miller WL (2010). Methods for evaluating practice change toward a patient-centered medical home. Ann Fam Med.

[69] Stacey CL, Henderson S, MacArthur KR, Dohan D (2009). Demanding patient or demanding encounter?: a case study of a cancer clinic. Soc Sci Med.

[70] Dohan D (2002). Managing indigent care: a case study of a safety-net emergency department. Health Serv Res.

[71] Sinding Christina (2010). Using Institutional Ethnography to Understand the Production of Health Care Disparities. Qualitative Health Research.

[72] Wind G (2008). Negotiated interactive observation: doing fieldwork in hospital settings. Anthropol Med.

[73] Jenkinson C, Coulter A, Bruster S (2002). The picker patient experience questionnaire: development and validation using data from in-patient surveys in five countries. Int J Qual Health Care.

[74] Jenkinson C, Coulter A, Reeves R, Bruster S, Richards N (2003). Properties of the picker patient experience questionnaire in a randomized controlled trial of long versus short form survey instruments. J Public Health Med.

[75] Rathert Cheryl, Williams Eric S., McCaughey Deirdre, Ishqaidef Ghadir (2012). Patient perceptions of patient-centred care: empirical test of a theoretical model. Health Expectations.

[76] Gustafson DH, Hawkins R, McTavish F, Pingree S, Chen WC, Volrathongchai K (2008). Internet-based interactive support for Cancer patients: are integrated systems better?. J Commun.

[77] Jeppesen KM, Coyle JD, Miser WF (2009). Screening questions to predict limited health literacy: a cross-sectional study of patients with diabetes mellitus. Ann Fam Med.

[78] Sarkar U, Fisher L, Schillinger D (2006). Is self-efficacy associated with diabetes self-management across race/ethnicity and health literacy?. Diabetes Care.

[79] Stiggelbout AM, de Haes JC, Vree R, van de Velde CJ, Bruijninckx CM, van Groningen K (1997). Follow-up of colorectal cancer patients: quality of life and attitudes towards follow-up. Br J Cancer.

[80] Hung DY, Glasgow RE, Dickinson LM, Froshaug DB, Fernald DH, Balasubramanian BA (2008). The chronic care model and relationships to patient health status and health-related quality of life. Am J Prev Med.

[81] Lerman CE, Brody DS, Caputo GC, Smith DG, Lazaro CG, Wolfson HG (1990). Patients' perceived involvement in care scale: relationship to attitudes about illness and medical care. J Gen Intern Med.

[82] Bishop WP, Lee SJC, Skinner CS, Jones TM, McCallister K, Tiro JA (2016). Validity of single-item screening for limited health literacy in English and Spanish speakers. Am J Public Health.

[83] Pastor R, Guallar E (1998). Use of two-segmented logistic regression to estimate change-points in epidemiologic studies. Am J Epidemiol.

[84] Borowsky SJ, Nelson DB, Fortney JC, Hedeen AN, Bradley JL, Chapko MK (2002). VA Community-based outpatient clinics - performance measures based on patient perceptions of care. Med Care.

[85] Cleary PD, Edgmanlevitan S, Roberts M, Moloney TW, Mcmullen W, Walker JD (1991). Patients evaluate their hospital-care - a National Survey. Health Aff.

[86] Wolf A, Olsson LE, Taft C, Swedberg K, Ekman I (2012). Impacts of patient characteristics on hospital care experience in 34,000 Swedish patients. BMC Nurs.

[87] Crabtree BF, Nutting PA, Miller WL, McDaniel RR, Stange KC, Jaen CR (2011). Primary care practice transformation is hard work: insights from a 15-year developmental program of research. Med Care.

[88] Simons L, Lathlean J, Squire C (2008). Shifting the focus: sequential methods of analysis with qualitative data. Qual Health Res.

[89] Farmer T, Robinson K, Elliott SJ, Eyles J (2006). Developing and implementing a triangulation protocol for qualitative Health Research. Qual Health Res.

[90] Thurmond VA (2001). The point of triangulation. J Nurs Scholarsh.

[91] Mays N, Pope C (1995). Rigour and qualitative research. BMJ.

[92] Cook TD, Shadish WR, Wong VC (2008). Three conditions under which experiments and observational studies produce comparable causal estimates: new findings from within-study comparisons. J Policy Anal Manag.

[93] Fereday J, Muir-Cochrane E (2006). Demonstrating rigor using thematic analysis: a hybrid approach of inductive and deductive coding and theme development. Int J Qual Methods.

[94] Miles MB, Huberman AM (1994). Qualitative data analysis.

[95] Morse JM (2004). Constructing qualitatively derived theory: concept construction and concept typologies. Qual Health Res.

[96] Cohen DJ, Crabtree BF (2008). Evaluative criteria for qualitative research in health care: controversies and recommendations. Ann Fam Med.

[97] Bradley EH, Curry LA, Devers KJ (2007). Qualitative data analysis for health services research: developing taxonomy, themes, and theory. Health Serv Res.

[98] Tinetti ME, Bogardus ST, Agostini JV (2004). Potential pitfalls of disease-specific guidelines for patients with multiple conditions. N Engl J Med.

[99] Bickell NA, Young GJ (2001). Coordination of care for early-stage breast cancer patients. J Gen Intern Med.

[100] Alsamarai Susan, Yao Xiaopan, Cain Hilary C., Chang Bryan W., Chao Herta H., Connery Donna M., Deng Yanhong, Garla Vijay N., Hunnibell Laura S., Kim Anthony W., Obando J. Antonio, Taylor Caroline, Tellides George, Rose Michal G. (2013). The Effect of a Lung Cancer Care Coordination Program on Timeliness of Care. Clinical Lung Cancer.

[101] Hershman DL, Greenlee H, Awad D, Kalinsky K, Maurer M, Kranwinkel G (2013). Randomized controlled trial of a clinic-based survivorship intervention following adjuvant therapy in breast cancer survivors. Breast Cancer Res Treat.

[102] Institute of Medicine (2013). Delivering High-Quality Cancer Care: Charting a new course for a system in crisis.

[103] Nekhlyudov L, Greene SM, Chubak J, Rabin B, Tuzzio L, Rolnick S (2013). Cancer research network: using integrated healthcare delivery systems as platforms for cancer survivorship research. J Cancer Surviv.

[104] Guidry JJ, Torrence W, Herbelin S (2005). Closing the divide: diverse populations and cancer survivorship. Cancer.

[105] Chubak J, Tuzzio L, Hsu C, Alfano CM, Rabin BA, Hornbrook MC (2012). Providing care for cancer survivors in integrated health care delivery systems: practices, challenges, and research opportunities. J Oncol Pract.

[106] Pollack LA, Hawkins NA, Peaker BL, Buchanan N, Risendal BC (2011). Dissemination and translation: a frontier for Cancer survivorship research. Cancer Epidemiol Biomark Prev.

[107] Wagner E, Austin B, Davis C, Hindmarsh M, Schaefer J, Bonomi A (2001). Improving chronic illness care: translating evidence into action. Health Aff (Millwood).

[108] Liss DT, Chubak J, Anderson ML, Saunders KW, Tuzzio L, Reid RJ (2011). Patient-reported care coordination: associations with primary care continuity and specialty care use. Ann Fam Med..

[109] MacPhail LH, Neuwirth EB, Bellows J (2009). Coordination of diabetes care in four delivery models using an electronic health record. Med Care.

[110] Long A, Bailit M (2010). Health reform and the patient-centered medical home: policy provisions and expectations of the patient protection and affordable care act.

[111] Witgert K, Hess C (2012). Including safety-net providers in integrated delivery systems: issues and options for policymakers. Issue Brief Commonw Fund.

[112] Neuhausen Katherine, Spivey Michael, Kellermann Arthur L. (2013). State Politics and the Fate of the Safety Net. New England Journal of Medicine.

[113] Graves JA (2012). Medicaid expansion opt-outs and uncompensated care. N Engl J Med.

[114] Maciejewski ML, Curtis LH, Dowd B (2013). Study design elements for rigorous quasi-experimental comparative effectiveness research. J Comp Eff Res.

[115] Harris AD, McGregor JC, Perencevich EN, Furuno JP, Zhu J, Peterson DE (2006). The use and interpretation of quasi-experimental studies in medical informatics. J Am Med Inf Assoc.

[116] Shadish WR, Cook TD, Campbell DT (2002). Experimental and quasi-experimental designs for generalized causal inference.

[117] Bayliss EA, Balasubramianian BA, Gill JM, Stange KC (2014). Perspectives in primary care: implementing patient-centered care coordination for individuals with multiple chronic medical conditions. Ann Fam Med.

[118] Kotronoulas G, Kearney N, Maguire R, Harrow A, Di Domenico D, Croy S (2014). What is the value of the routine use of patient-reported outcome measures toward improvement of patient outcomes, processes of care, and health service outcomes in Cancer care? A systematic review of controlled trials. J Clin Oncol.

[119] Hacker K, Santos P, Thompson D, Stout SS, Bearse A, Mechanic RE (2014). Early experience of a safety net provider reorganizing into an accountable care organization. J Health Polit Policy Law.

